# Effectiveness of cognitive behavioral therapy for hypertension-related psychological distress: a two-phase randomized controlled trial in a Nigerian tertiary hospital

**DOI:** 10.3389/fpsyg.2025.1684461

**Published:** 2026-01-12

**Authors:** Addah Temple Tamuno-opubo, Wandile F. Tsabedze, Curwyn Mapaling, Egbewuare Emmanuel Idehen, Jamiu Tinuoye Uthman

**Affiliations:** 1 Department of Psychology, Faculty of Social Science, Olabisi Onabanjo University, Ago-Iwoye, Ogun, Nigeria; 2 Department of Psychology, University of South Africa, Pretoria, South Africa; 3 Optentia Research Unit, North-West University, Potchefstroom, South Africa; 4 Department of Psychology, Faculty of Social Science, Obafemi Awolowo University, Ile-Ife, Osun, Nigeria

**Keywords:** behavioral medicine, chronic disease, cognitive behavioral therapy, hypertension, mental health, non-pharmacological interventions, psychological distress, randomized controlled trial

## Abstract

**Background:**

Hypertension is a major contributor to cardiovascular morbidity globally and is frequently accompanied by psychological distress, including anxiety and depression. Evidence on the efficacy of psychological interventions, particularly Cognitive Behavioral Therapy (CBT), for hypertension-related distress remains limited in the Sahara South of Africa.

**Objective:**

This study examined the effectiveness of an 8-week CBT program in reducing psychological distress among adults with hypertension attending a Nigerian tertiary hospital, compared with a waitlist/usual care control group.

**Methods:**

A two-phase randomized controlled trial was conducted. In Phase I, 242 hypertensive patients were screened for psychological distress. Phase II involved 42 participants with moderate to severe distress (mean age = 44.9 years) who were randomly assigned to either an 8-week CBT intervention or a waitlist control, continuing usual hypertension care. Psychological distress was assessed using the Symptom Checklist-13 (SCL-13). Data were analyzed per protocol using paired and independent *t*-tests and ANCOVA, controlling for baseline distress and age.

**Results:**

The CBT group showed significant reductions in distress from baseline (M = 13.78, SD = 3.04) to post-test (M = 9.45, SD = 2.31), *t*_(20)_ = 8.12, *p* < 0.001, whereas the control group showed no meaningful change (baseline M = 13.42, SD = 2.92; post-test M = 13.21, SD = 2.85), *t*_(20)_ = 0.48, *p* = 0.630. Between-group comparisons confirmed greater improvement in the CBT group, *t*_(40)_ = 5.64, *p* < 0.001, Hedges' g = −1.42. Subgroup analyses revealed large effects for participants with severe [*t*_(9)_ = 5.85, *p* < 0.001, partial η^2^ = 0.62] and moderate distress [*t*_(10)_ = 4.42, *p* = 0.012, partial η^2^ = 0.48]. Age significantly influenced outcomes, *F*_(1, 40)_ = 5.24, *p* = 0.040, while gender and marital status did not.

**Conclusion:**

These findings provide evidence that an 8-week CBT intervention significantly reduces psychological distress in individuals with hypertension. The results support the integration of CBT into hypertension care within Nigerian tertiary hospitals, highlighting the potential for broader implementation in similar clinical settings. Future research, involving larger and longer-term studies, is recommended to confirm the durability and generalizability of these findings.

## Introduction

High blood pressure, or hypertension (HTN), is one of the leading health problems worldwide and a major cause of cardiovascular disease, stroke, and premature death (Fuchs and Whelton, 2020; Mills et al., 2020; World Health Organization, 2023a). It represents a growing global concern, with more than one billion people currently estimated to be living with the condition (Mills et al., 2020). This burden is especially severe in developing regions, where limited access to preventive care and early diagnosis contributes to higher prevalence rates. In Nigeria and other low- and middle-income countries, between 30% and 45% of adults are hypertensive (Schutte et al., 2021; Adeloye et al., 2021), highlighting the magnitude of the public health challenge. Although progress has been made in pharmacological management and lifestyle modification, these approaches often overlook the psychological dimension of hypertension. The emotional burden associated with hypertension, particularly anxiety, depression, and psychological distress (PD), has received limited attention in clinical practice (Ojike et al., 2016; Kretchy et al., 2014). This neglect is critical because mental wellbeing strongly influences treatment adherence, medication consistency, and long-term recovery. However, psychosocial care remains a largely neglected aspect of hypertension management in many healthcare systems, including Nigeria (Levine et al., 2021; Pawel and Natalia, 2024; World Health Organization, 2019; Fadele et al., 2024).

The expression of the psychological distress in hypertension takes emotional and behavioral forms which include anxiety, depression, somatization, obsessive-compulsive symptoms/character and increased neuroticism. These psychological determinants, when not controlled, can increase the burden of disease, reduce compliance to treatment and eventually lead to a poor clinical outcome (Ojike et al., 2016; Alwani et al., 2023). Such distress, over time, not only worsens the ability of patients to cope with their health condition in a productive manner but also reduces the overall quality of life of the patients (Fatima et al., 2025). With such implications, there is an urgent need to incorporate psychological interventions as a standard practice in hypertension management. Among the evidence-based and well-established techniques, Cognitive Behavioral Therapy (CBT) has been found to be effective in the alleviation of distress, anxiety and depression amongst individuals with chronic illness (Wilson and Gil, 1996; Day, 2012; Lunkenheimer et al., 2020). The systematic and goal-oriented nature of Cognitive Behavioral Therapy helps patients to understand their maladaptive thinking patterns and change them to build more adaptive coping and emotional control systems (Vela and Carroll, 2023). In addition to the psychological benefits, emerging evidence suggests that CBT may also have physiological benefits, including reduced stress and increased emotional resilience, which are central to the management of hypertension (Tabibnia and Radecki, 2018; Ghasemi et al., 2024).

Although the efficacy of CBT in managing hypertension-related psychological distress is well-documented across different global settings, its utilization in the management of hypertension-related psychological distress has not been common in low-resource healthcare settings. This disconnect is especially obvious in Nigeria, where mental health is limited due to stigma, a lack of qualified personnel, and limited funds. In this case, CBT can be described as a culturally flexible and pragmatic intervention that may be incorporated into current chronic disease management models (Boroń-Krupińskaabde and Kulmatyckicde, 2016; Carlson et al., 2025). Nonetheless, the general management of hypertension in Nigeria is at a very worrying level. The 2015 national hypertension surveillance data show that approximately 13 percent of treated patients have an adequate level of blood pressure control across various community and hospital programmes (Ojji et al., 2022; World Health Organization, 2023b). Such statistics support the urgency of complementary, evidence-based practices, including psychological interventions, to reinforce comprehensive disease management. The Nigerian government is aware of this challenge, and therefore, the selection of the National Multi-Sectoral Action Plan for the Prevention and Control of Non-Communicable Diseases (2019–2025) aligns with global targets, including Sustainable Development Goal 3 (Good Health and Wellbeing) [International Cancer Control Partnership (ICCP), 2025]. The plan will entail novel, combined approaches that integrate physical and mental health care within existing systems, supporting the rationale of this study. Consideration of the effectiveness of CBT in such environments is hence vital toward facilitating holistic, patient-focused treatment of hypertension. The CBT 8-week model implemented in this paper was based on the following priorities and was informed by internationally recognized forms of brief therapy that combine clinical effectiveness with cost-effectiveness, making it particularly applicable in the tertiary hospital setting with limited resources.

Sociodemographics in terms of age, sex and marital status had demonstrated significant effects on psychological outcomes and affected how individuals responded to treatment intervention (Gureje et al., 2010; Ogunwale et al., 2023; Radhi et al., 2023). These variables are not independent, and factors are multidetermined, involving coping styles and engagement with therapy. For instance, older adults may use coping strategies that are different from those of the younger population (Knight and Satre, 1999; Saunders et al., 2021), which might influence their response to CBT treatment. Similarly, there may be gender-based differences in the expression and help-seeking behaviors of emotions. Marital status dictates the availability of social and emotional support for people living with chronic diseases (Adzrago et al., 2024). Given that treatment response rates have not improved in decades, such findings are important for understanding heterogeneity in therapeutic response and developing interventions that are responsive to individual differences and the contexts within which individuals live.

Considering this, the current research was conducted at the Obafemi Awolowo University Teaching Health Center (OAUHC), Ile-Ife, Nigeria, a mini tertiary hospital rendering primary and specialist care. The hospital caters to a mixed patient group across the local community, and thus it is an ideal environment to carry out the psychological intervention testing in the hypertension departments (Awotidebe et al., 2017, 2014; Ogunyemi et al., 2023). This situation offered a natural contextual setting to evaluate the clinical effectiveness of CBT in a natural Nigerian tertiary care setting. The research was informed by the Cognitive Theory of Psychological Distress, which postulates that emotional distress is not only caused by external events in life, but also by the way individuals think and interpret these external events as cognition (Beck, 2019). In this framework, poor or distorted thinking patterns, including hopelessness, catastrophizing, or self-blame, may increase emotional suffering and reduce compliance with treatment. Cognitive Behavioral Therapy is the direct method of correcting these cognitive distortions, as it allows individuals to recognize, dispute and substitute maladaptive thoughts with more balanced and adaptive ones. By doing this, patients can be more emotionally controlled and less distressed, as well as have healthier behavioral patterns that would be able to sustain their hypertension in the long term (Hofmann et al., 2012; Cuijpers et al., 2016; Isa et al., 2018).

### Study aim

This study aimed to evaluate the effectiveness of an 8-week Cognitive Behavioral Therapy (CBT) programme (Intervention) compared with waitlist/usual care (Comparison) in reducing psychological distress (Outcome) among adults with hypertension (Population) receiving outpatient care at the Obafemi Awolowo University Teaching Hospitals Complex, Ile-Ife, Nigeria (Setting) over an 8-week period (Time).

### Specific objectives

1. Ascertain the effectivness of cognitive behavioral therapy in the management of psychological distress among people living with hypertension; and
2. Examine the influence of sociodemographic factors (such as age, gender, and marital status) on the effectiveness of CBT in reducing PD among people living with hypertension.

### Research hypotheses

Null Hypothesis (H_01_): An 8-week Cognitive Behavioral Therapy (CBT) intervention will have no significant effect on the psychological distress of people living with hypertension.

Alternate Hypothesis (H_11_): An 8-week Cognitive Behavioral Therapy (CBT) intervention will significantly reduce the psychological distress of people living with hypertension.

Null Hypothesis (H_02_): Sociodemographic variables (age, gender, and marital status) will have no significant influence on the effectiveness of CBT in reducing psychological distress among people living with hypertension.

Alternate Hypothesis (H_12_): Sociodemographic variables (age, gender, and marital status) will have a significant impact on the effectiveness of CBT in reducing psychological distress among individuals living with hypertension.

## Methods

This study employed a two-phase randomized controlled trial (RCT) design. A descriptive survey design was employed in the first phase, which involved screening participants for psychological distress. This provided baseline data (or the pre-test scores) for all the participants. The second phase adopted an RCT pre- and post-test experimental design. Participants at this stage were those who scored high (within the moderate and severe symptoms range) on psychological distress in the first phase. Participants were randomly assigned to either (a) the CBT intervention arm, receiving eight weekly sessions, or (b) a waitlist control arm. The waitlist group continued with usual hypertension care during the 8-week period and was subsequently offered CBT following completion of the study. This design ensured ethical balance while preserving a valid comparison group.

### Phase 1: screening

This subsection provides details of the methods used in Study Phase 1, including the research design, population, sample, sampling procedure, research instruments, data collection procedure, and data analysis.

### Research design

A cross-sectional descriptive survey design was used in this phase of the study. Primary data was collected through the administration of a set of standardized psychological scales (described in detail under the “Research Instruments” subsection) on a convenience sample of the study population.

### Sample and sampling procedure

The research was conducted at the Obafemi Awolowo University Health Center (OAUHC), Ile-Ife, Nigeria, a university-based primary and secondary care center that provides outpatient medical, nursing, and laboratory services to university personnel, students, and the surrounding communities. The clinic offers specific hypertension follow-up services, including regular check-ups, medication reviews, and lifestyle counseling, which is typical of a Nigerian tertiary-linked health center where chronic disease management is part of regular outpatient services. The study population included individuals with hypertension who were registered as such at the OAUHC; 1,655 registered hypertension patients from the hospital were present at the time of the study. A consecutive sample of 242 patients attending regular hypertension clinics (both held on Tuesdays and Wednesdays) at the clinic was screened using the SCL−13 (Symptom Checklist−13) (Tamuno-opubo, 2023) to represent possible accessibility within the study period. The majority of patients in this environment go there with silent hypertension that is only revealed during routine check-ups and not the actual symptoms; hence, attendance in this context was due to the psychological stress of having a chronic, asymptomatic illness. The convenience sampling was suitable here since eligible patients visited the clinic on various days and at various times, and enrolling the present patients was convenient. It is typical of clinic-based cross-sectional studies, where random sampling of attendees is not possible, in exploratory surveys of psychological distress.

The Phase I screening sample was pragmatically selected based on clinic attendance patterns and feasibility. The sample size targeted was 220 hypertensive patients, inflated by 10 per cent to account for potential participant loss and missing data, resulting in an overall target of approximately 242 patients. The methodology created sufficient coverage in identifying the prevalence of psychological distress in the clinic environment. *Post-hoc*, the prevalence of psychological distress in this sample was observed to be 50.8, with a 95% confidence interval of approximately 6.3, which is sufficient to achieve satisfactory precision in a feasibility screen. Notably, none of the subjects were in the ‘No PD' category (SCL−13 score 0–5).

For the final sample size calculation, the formula by Araoye (2004) was used:


$$\begin{array}{l} n=\frac{{\text{Z}}^{2*}\text{P}\left(1-\text{P}\right)}{{\text{d}}^{2}} \end{array}$$

From the formula given,

n = sample size

Z = the standard normal deviation set as 1.96, which is 95% confidence level

P = The prevalence of psychological distress among staff members of Obafemi Awolowo University, Ile-Ife, Osun State, was 17.6%, as used by Moughalu (2021), Southwestern Nigeria.

d = Degree of accuracy = 0.05

Hence, calculated thus:


$$\begin{array}{l} \text{n}=\frac{1.96^{2}\times 17.6\%\left(1-17.6\%\right)}{0.05^{2}}=\frac{0.5511\;}{0.0025\;}=220.43\sim \;220 \end{array}$$

Attrition rate: (calculate 10% of 220) and add to 220; following standard practice in survey research, where modest inflation is recommended to accommodate non-response and unusable questionnaires (Araoye, 2004).


$$\begin{array}{lll} 10\%\text{ of }220 & = & \;22\\ \text{Sample size} & = & 220+22=242\text{ Respondents}. \end{array}$$

The mean age of the 242 respondents was approximately 43 years, with a standard deviation of 15.79. The majority (*n* = 151, 62.4%) of the respondents were female, compared to 37.6% males. Likewise, most (75.60%) respondents were married, and (73.60%) were Christians. The majority (77.30%) of the respondents had a tertiary education, while (60.70%) had an average monthly income. This information is presented in Table 1.

**Table 1:** Socio-demographic characteristics of participants in the first phase of the study (*N* = 242).

**Variables**	**Groups**	**Frequency**	**Percentage (%)**
**Age: 42.91** ±**15.79**			
Gender	Male	91	37.60
	Female	151	62.40
Marital status	Married	183	75.60
	Single	39	16.10
	Divorced	4	1.70
	Separated	3	1.20
	Widower	13	5.4
Religion	Christianity	178	73.60
	Islam	59	24.40
	Others	5	2.10
Level of education	None	1	0.4
	Primary	2	0.8
	Secondary	52	21.50
	Tertiary	187	77.30
Monthly income	Low	82	33.90
	Average	147	60.70
	High	13	5.40

Author's source, 2023.

### Inclusion and exclusion criteria

#### Inclusion criteria

At OAUHC, hypertension was confirmed by general practitioners based on repeated elevated blood pressure readings over at least 1 month, sometimes supported by basic tests. For this study, inclusion was restricted to patients already formally diagnosed and under follow-up in the hypertensive clinic. This ensured that participants had a confirmed diagnosis and were already engaged in routine hypertension care, thereby reducing diagnostic ambiguity and controlling for acute medical instability that could confound psychological distress scores.

#### Exclusion criteria

Participants were excluded if they had any comorbidities, including psychiatric conditions or other chronic conditions such as diabetes or chronic respiratory diseases. Only individuals diagnosed with hypertension were eligible for participation. Additionally, individuals with elevated distress scores, regardless of whether they were previously diagnosed, were excluded if they did not meet the criteria for a hypertension diagnosis.

### Research instruments

This sub-section contains detailed information on the following psychological instruments used to collect data in this study phase.

**Bio-Data Form (BDF):** The BDF was developed by the researchers to describe the participants, drawing on socio-demographic items commonly used in Nigerian hypertension studies, such as age, gender, marital status, education, employment and income level, which have been included as measures of the independent variables.

**Psychological Distress:** The Psychological Distress (PD) measure used in the study was the Symptom CheckList (SCL), which comprises 13 items, originated by (Tamuno-opubo 2023). The SCL-13 was based on the Symptom Checklist (SCL-90) by Derogatis (1977), yet it was supposed to be shorter. (Tamuno-opubo 2023) developed and validated the SCL−13 as a measure to be utilized with people living with Hypertension in the Obafemi Awolowo University Health Center OAUHC). It was designed for use in routine outcome assessment, including session-by-session monitoring. The SCL comprises five domains: Somatization (items 1–3), Obsessive-Compulsive Behavior (Items 4–5), Depression (Items 6–8), Anxiety (Items 9–11) and Neuroticism (Items 12–13). Items on SCL−13 are phrased in the form of single words (symptoms), short phrases or sentences. Sample items include: “soreness of your muscle,” “pain in heart or chest,” and “thought of death or dying,” amongst others. Each item is scored on a scale of 0 to 4 based on how much an individual was bothered by each item in the last week, including the day the respondent completed the scale. Thus, each item carried the 5-point Likert-type response of 0 = “Not at all”; 1 = “A little bit”; 2 = “Moderately”; 3 = “Quite a bit”; 4 = “Extremely”. The overall values of the five sections are further combined to obtain the overall psychological distress score. Thus, total scores can range from zero to 52. To select participants for the second phase of the study and determine the level of psychological distress among the respondents, extreme scores, defined as those one standard deviation above and below the mean (that is, x ± 1SD), were used. In essence, the SCL−13 was used as a screening scale for psychological distress in Phase I (pre-test) and also as an evaluation scale in Phase II (post-test). The original SCL−90 has proven to possess adequate psychometric properties. For instance, the author reported a Cronbach's Alpha of 0.98. Another study reported a Cronbach's Alpha 0.92 (Ardakani et al., 2016). The SCL−13 has been used among people living with hypertension in Nigeria, with a Cronbach's Alpha score of 0.92 (Tamuno-opubo, 2023).

### Data collection procedure

To ensure a systematic approach, the researcher first obtained an introductory letter from the Department of Psychology, which was presented to the management of the Obafemi Awolowo University Health Center (OAUHC). In OAUHC, any research proposal must be reviewed and approved by a research committee before data collection can commence. Additionally, ethical approval was sought from the Research Ethics Committee of the Department of Public Health at Obafemi Awolowo University, with clearance granted under HREC NO: IPH/OAU/12/2130. After that, the researchers were offered the opportunity to collaborate with key stakeholders, such as consultants, nurses, and other healthcare professionals in the corresponding departments, which contributed to the promotion of collaboration and the successful identification and enrolment of the necessary group of participants in the research. This trial was not prospectively registered.

The data was collected in the hospital during the working days (Tuesday and Wednesday), which are clinic days. People who had been waiting to be seen by medical personnel within the hospital were assisted during the waiting period before seeing doctors and later filling prescriptions at the drugstores. To make the process more convenient, the researchers established a rapport with the patients in the waiting room and obtained their consent to participate in the research. They then directed them to complete the research questionnaire. In such a way, individuals with moderate and severe psychological distress, according to SCL-13-SF (Tamuno-opubo, 2023), could be followed up and used in the next stage of the research. In order to streamline the data collection process, professional research assistants, who were also employees of the health center, were hired to facilitate the process. The screening instruments were assembled into one research protocol, and instructions were read to the participants so that they were clear. To ensure anonymity, a designated drop box was located in the waiting room, where participants could leave their completed questionnaires. Nevertheless, those people who wanted to give their responses personally to the researcher could choose this way. After completion, the researcher collected all the questionnaires to ensure that there were no gaps in the data.

### Data analysis

Data collected in the study were analyzed using descriptive statistics, such as mean, standard deviation, frequency, and percentage counts, to describe the respondents and aggregate the data, providing participants for the second phase of the study. These analyses were carried out using the IBM SPSS Version 25.0 sub-programmes.

### Phase II: management

This subsection contains details of the research design, population, sample, and sampling technique, as well as the research instruments and data collection and analysis procedures employed in Phase II.

### Research design

A total of 42 participants were recruited for the second phase and randomly allocated into an intervention group (*n* = 21) and a waitlist control group (*n* = 21). This phase employed a randomized controlled trial with a pre-test–post-test design. Psychological distress was measured in both groups before the intervention (pre-test) and after the 8-week programme (post-test), allowing assessment of changes attributable to CBT.

### Study population

The population for the intervention phase consisted of people living with hypertension who scored high on the SCL−13 in the survey phase of the study. Cut-offs for categorizing psychological distress were based on SCL−13 total scores: No PD (0–5), Mild (6–21), Moderate (22–37), Severe (38–52). Cut-offs for categorizing psychological distress were based on SCL-13 total scores as suggested in the scale development work (Tamuno-opubo, 2023).

### Sampling and sampling technique

No formal power calculation was conducted for Phase II; the sample size of 42 (21 per group) was determined pragmatically in line with pilot RCT conventions. Participants with moderate to severe psychological distress were purposively selected and then randomly assigned to either the CBT intervention group (n = 21) or the waitlist control group (n = 21). The intervention group received 8 weeks of structured CBT sessions, while the control group continued with usual antihypertensive care and routine consultations at the OAUHC during the study, with CBT offered after trial completion. Importantly, refusal to participate in CBT did not determine allocation; group assignment followed an independently managed odd–even allocation list to minimize bias.

The sociodemographic characteristics of the participants who met the inclusion criteria for this phase of the study are presented in Table 2. The mean age of the participants was 44.91 ± 19.90 years. Most participants (59.52%) were female, and all were married. Most (83.33%) of the participants were Christians; the majority had tertiary (post-secondary) education, such as a diploma or university degree (85.7%), with 14.3% having completed secondary education. Likewise, most (71.60%) of them had an average level of monthly income.

**Table 2:** Baseline socio-demographic characteristics of Phase II participants by allocation group (CBT vs. control) (*N* = 42).

**Variable**	**CBT group (*n* = 21)**	**Control group (*n* = 21)**	**Total (*N* = 42)**
Age (mean ± SD)	45.2 ± 20.1	44.6 ± 19.8	44.9 ± 19.9
**Gender**			
Male	8 (38.1%)	9 (42.9%)	17 (40.5%)
Female	13 (61.9%)	12 (57.1%)	25 (59.5%)
**Marital status**			
Married	21 (100%)	21 (100%)	42 (100%)
**Religion**			
Christianity	18 (85.7%)	17 (81.0%)	35 (83.3%)
Islam	3 (14.3%)	4 (19.0%)	7 (16.7%)
**Education level**			
Secondary	3 (14.3%)	3 (14.3%)	6 (14.3%)
Tertiary	18 (85.7%)	18 (85.7%)	36 (85.7%)
**Monthly income**			
Low	3 (14.3%)	3 (14.3%)	6 (14.3%)
Average	15 (71.4%)	15 (71.4%)	30 (71.4%)
High	3 (14.3%)	3 (14.3%)	6 (14.3%)

CBT, intervention group; Control, waitlist/usual care group. Values are presented as mean (SD) or *n* (%). Independent *t*-tests (for continuous variables such as age and baseline distress scores) and Chi-square tests (for categorical variables) were performed to assess baseline equivalence between groups. Where expected cell frequencies were < 5, Fisher's Exact Test was applied to ensure statistical validity. No significant baseline differences were observed between the CBT and control groups across socio-demographic variables (all *p* > 0.05).

As shown in Table 2, the socio-demographic characteristics of participants in the CBT and control groups were comparable at baseline, with no statistically significant differences across age, sex, education, income, or marital status. Monthly income was recorded in three pre-specified brackets: low (< ₦70,000), average (< ₦71,000– < ₦150,000), and high (>₦150,000), as operational categories for descriptive comparison.

**Inclusion and Exclusion Criteria:** Participants in this phase of the study met the cut-off scores for moderate and severe symptoms of PD and consented to participate in the therapy process. Patients who declined participation in CBT were excluded entirely from Phase II and were not placed in the control arm. This approach avoided contamination of the control group with participants who had negative expectations toward psychotherapy.

### Research instrument

Only the SCL−13 (described in phase 1) was employed in this phase of the study.

### Treatment and data collection procedure

The study's first phase involved documenting participants' names, phone numbers, and file numbers. This was to avoid the problem of losing contact with respondents. Only participants who met the criteria set in phase 1 were included in this phase of the study. The odd–even allocation list was generated and managed independently, and the sequence was locked prior to participant enrollment, preventing participants or treating clinicians from gaining foreknowledge of their assignment. The group assignment was revealed only after the baseline assessment was completed, and enrollment was finalized.

**Session 1:** During the first day's visit to the clinic, all participants were involved in a focus group discussion (FGD), where factors associated with their psychological distress were identified and incorporated into the treatment modules. After the FGD, participants were randomly assigned to the treatment (treatment with CBT) or control (no-intervention) groups based on the odd-even numbers in the sampling frame, which was a list of all participants in each category. Participants were randomly assigned to either the CBT or control groups using a pre-prepared odd–even allocation list, which was held by an independent administrator who was not involved in treatment delivery or data collection. This ensured that allocation was concealed from both participants at the point of enrolment and from researchers delivering the intervention. Participants in the control group only participated during the first session. Only the intervention group received intervention training courses on CBT for eight (8) weekly sessions, each of which lasted for about 90 min on Wednesdays. The CBT sessions were delivered as group therapy by a licensed clinical psychologist trained using a structured CBT manual adapted for people living with hypertension. The psychologist received weekly supervision to ensure therapeutic fidelity throughout the intervention. “The intervention was based on Beck's Cognitive Behavioral Therapy manual (Beck, 2019), which was adapted to fit the cultural and clinical context of hypertensive patients in Southwestern Nigeria.” These sessions were held in the morning, from 10 a.m. to 11:30 a.m. A therapist checklist and session logs were used during each intervention to maintain consistency and ensure treatment fidelity across sessions. Sessions were also periodically reviewed under supervision. They were carried out in the CBT approach as follows:

**Session 2:** Assessment or psychological assessment—this was used to identify maladaptive feelings and thoughts through the use of exposure techniques (oriented the patients to CBT; assessed their concerns, and set initial treatment plan/goals).

**Session 3:** Reconceptualization—This was used to address the most distressing psychological symptom among the participants (assessment of patients' concerns continued, setting goals continued, and the beginning of intervention).

**Session 4:** Skills Acquisition—This helped participants take responsibility for their behavior, gain self-respect, validate themselves and their emotions, reduce interpersonal problems, and move toward attaining goals (continuation of intervention).

**Session 5:** Skills Consolidation and Application Training—This session was used to enhance the capacity for more joy in life alongside a sense of wellbeing (continuation of intervention; evaluation of goals/treatment plan).

**Session 6:** Generalization and maintenance—The therapist and clients discussed the future and how the clients will cope once they have left treatment (continuation of intervention; discussion on ending treatment, and prepared patients on how to manage and maintain changes).

**Session 7:** Post-treatment assessment and termination—Post-intervention assessments were completed and participants were supported to consolidate treatment gains. Plans for maintaining coping strategies and relapse prevention were discussed (termination of active intervention; maintenance of treatment gains).

After the post-intervention assessments were completed, both the intervention and control groups were invited to a structured debriefing session. This provided participants with psychological support, clarified the study procedures, and addressed any lingering concerns. Participants in the control group received usual care during the study period and were offered CBT after the study completion. This ensured that no participants were deprived of the intervention.

### Intervention procedure and compliance

All participants in the CBT group received the full 8-week intervention, which consisted of weekly individual sessions. The CBT program was delivered by trained therapists, and adherence was closely monitored throughout the study. All participants in the CBT group completed the full intervention. No participants were excluded due to non-compliance, and 100% adherence was reported across all sessions.

### Analysis of data

Data collected in this phase of the study were analyzed using both descriptive and inferential statistics. Descriptive statistics, including means, standard deviations, frequencies, and percentages, were used to describe the participants and aggregate the data. The results were presented in the form of Figures and Tables. Assumptions of normality and homogeneity of variance were checked using the Shapiro-Wilk test and Levene's test, respectively. Paired *t*-tests were used to examine within-group changes from pre- to post-intervention, while independent *t*-tests were employed to compare post-intervention scores between the intervention and control groups. The data were analyzed using Analysis of Covariance (ANCOVA) to examine post-intervention differences while controlling for sociodemographic variables such as age (with age entered as a categorical variable), gender, and marital status. However, marital status was found to be non-significant in the preliminary analyses and was excluded from the final model. For variables with expected frequencies less than 5, Fisher's Exact Test was applied instead of the Chi-Square test to ensure accurate interpretation of categorical data. Analyses were conducted on a per-protocol basis (participants with completed pre- and post-intervention data); intention-to-treat analyses were not performed and are noted as a limitation. All tests were two-tailed, and significance was set at p < 0.05. The analyses were conducted using IBM SPSS Version 25.0.

## Results

The goal here was to determine the prevalence of psychological distress among people living with hypertension at Obafemi Awolowo University Health Center, Ile-Ife. Psychological distress was categorized as Severe, Moderate and Mild based on the x ± SD criterion. The standard deviation (16.28) was subtracted from or added to the mean (20.92). The lower cut-off is 20.92–16.28 = 4.64, approximately a score of five (5). The upper cut-off was 20.92 + 16.28 = 37.20, approximately a score of 37. The score range on SCL−13 (the measure of PD was zero [0] to 52. The respondents in the range of 0–5 were categorized as having no PD (Normal); those with scores in the range of 6–21 were classified as having mild symptoms of PD; those whose scores fell between 22 and 37 were categorized as having moderate symptoms of PD, while those with scores of 38 and above were categorized as having severed PD. The results of this analysis are presented in Table 3.

**Table 3:** Level of psychological distress among respondents.

**Variable category**	**Score range**	**Frequency**	**Percentage**
Mild	6–21	119	49.20
Moderate	22–37	60	24.70
Severe	38 and above	63	26.10

No participants were classified as “No PD” (0–5 on SCL−13).

**Figure 1:**
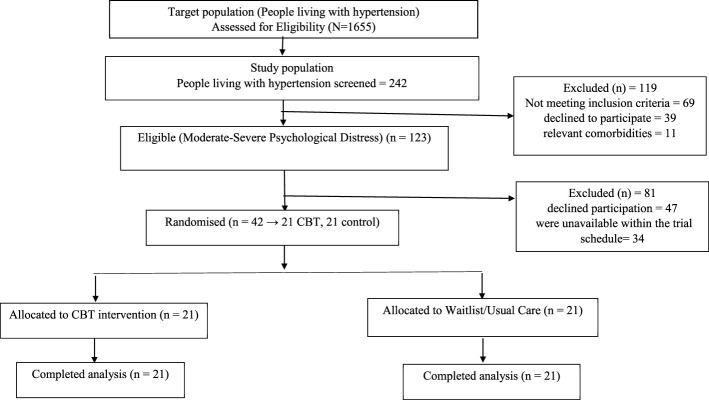
Research Design as per the CONSORT guidelines, 2010. CBT, intervention; waitlist/usual care, standard antihypertensive care with routine consultations, no psychological intervention during the study; CBT was offered to controls after completion. Flow of participants through the study (as per CONSORT 2010): 1,655 assessed → 242 screened → 123 eligible → 42 randomized (21 CBT, 21 wait-list control).

Table 3 also indicated that upon the first screening, 26.10% of the respondents had severe psychological distress, while 24.70% had moderate distress. In total, 50.8% of the participants experienced psychological distress (severe and moderate), with the remaining 49.20% classified as having mild distress. These formed the basis for selecting participants for the second phase of the study, as illustrated in Figure 1 below:

**Hypothesis One:** This hypothesis stated that cognitive behavioral therapy will not be significantly effective in the management of psychological distress among people living with hypertension. The hypothesis was tested with a series of *t*-tests for related measures. The results of the analysis are presented in Tables 4–6.

**Table 4:** Paired-samples *t*-test comparison of mean pre-test and post-test SCL-13 scores on psychological distress.

**Group**	**Time point**	***n***	***M***	***SD***	***df***	***t***	***p***	***η^2^***
Severe exp A	Pre-test	10	44.20	3.10	9	5.85	< 0.001	0.62
	8th assessment	10	28.10	4.20	–	–	–	–
Moderate exp B	Pre-test	11	30.50	2.80	10	4.42	0.012	0.48
	8th assessment	11	18.70	3.00	–	–	–	–
Severe CTRL A	Pre-test	10	43.50	3.50	9	1.20	0.26	–
	8th assessment	10	42.80	3.70	–	–	–	–
Moderate CTRL B	Pre-test	11	31.20	3.00	10	0.75	0.47	–
	8th assessment	11	30.80	3.10	–	–	–	–

All values represent paired-samples *t*-tests comparing pre-test and 8th-session SCL-13 total scores (range 0–52). exp A, severe CBT group; exp B, moderate CBT group; CTRL A, severe control group; CTRL B, moderate control group; CBT, Cognitive Behavioral Therapy; SCL-13, Symptom Checklist-13. Intervention groups received 8 weeks of CBT; control groups received usual antihypertensive care (waitlist/usual care). Effect sizes (η^2^) are reported for the CBT groups, where large reductions in distress were observed.

**Table 5:** Pre-test and post-test scores for cognitive behavioral therapy (CBT) and control groups.

**Group**	**Pre-test score (mean ±SD)**	**Post-test score (mean ±SD)**	**change in score (Mean)**
Severe control	43.50 ± 3.50	42.80 ± 3.70	−0.70
Moderate control	31.20 ± 3.00	30.80 ± 3.10	−0.40
CBT group	39.20 ± 4.10	30.50 ± 4.50	−8.70

Pre- and post-intervention SCL-13 total scores (range: 0–52). The CBT group received the intervention, while the control group (waitlist/usual care) did not. Paired *t*-tests were used to assess within-group changes, and an independent *t*-test was conducted to compare post-test scores between groups. For the control group, the pre–post change was non-significant [*t*_(20)_ = 0.48, *p* = 0.630]. The between-group comparison at post-test showed a significant difference [*t*_(40)_ = 5.64, *p* < 0.001], indicating that the CBT group experienced a significant reduction in psychological distress compared to the control group.

**Table 6:** Paired *t*-tests for pre–post change in CBT subgroups (severe and moderate).

**Groups**	**Variables**	***N***	**Pre-test M (SD)**	**Post-test M (SD)**	**Mean change**	***t(df)***	***p***	***η^2^***
Severe _expA_	8th assessment	10	44.20 (3.10)	28.10 (4.20)	−16.10	5.85 (9)	< 0.001	0.62
Moderate _expB_	8th assessment	11	30.50 (2.80)	18.70 (3.00)	−11.80	4.42 (10)	< 0.001	0.48

Within-group paired *t*-tests for CBT participants only; SCL-13 total scores (0–52). Severe: *t*_(9)_ = 5.85, *p* < 0.001; Moderate: *t*_(10)_ = 4.42, *p* = 0.012.

The results of the paired-samples *t*-tests (Table 4) show that, among participants with severe psychological distress in the CBT group, the pre-test mean SCL-13 score was 44.20 (SD = 3.10), which decreased to 28.10 (SD = 4.20) at the 8th assessment, *t*_(9)_ = 5.85, *p* < 0.001, η^2^ = 0.62, indicating a large and statistically significant reduction in distress. For participants with moderate psychological distress in the CBT group, the pre-test mean was 30.50 (SD = 2.80), declining to 18.70 (SD = 3.00) post-intervention, *t*_(10)_ = 4.42, *p* = 0.012, η^2^ = 0.48, also reflecting a substantial treatment effect.

In contrast, the waitlist control groups did not show a significant change over time. The severe control group had a pre-test mean of 43.50 (SD = 3.50) and a post-test mean of 42.80 (SD = 3.70), *t*_(9)_ = 1.20, *p* = 0.26, while the moderate control group moved only from 31.20 (SD = 3.00) to 30.80 (SD = 3.10), *t*_(10)_ = 0.75, *p* = 0.47. These small, non-significant fluctuations suggest that usual care alone did not meaningfully reduce psychological distress. Overall, the pattern of results indicates that CBT was effective in reducing psychological distress among people with hypertension in both the severe and moderate intervention groups compared with the waitlist controls. Effect size estimates show that approximately 62% of the variance in distress reduction was attributable to CBT in the severe group (η^2^ = 0.62) and 48% in the moderate group (η^2^ = 0.48), underscoring the clinical impact of the intervention.

As shown in Table 5, participants in the CBT intervention group experienced a significant reduction in psychological distress over the 8-week period. The mean score decreased from 13.78 (SD = 3.04) at baseline to 9.45 (SD = 2.31) post-intervention, a change that was statistically significant, *t*_(20)_ = 8.12, *p* < 0.001. In contrast, the control group (waitlist/usual care) showed minimal change, with scores of 13.42 (SD = 2.92) at baseline and 13.21 (SD = 2.85) post-test, *t*_(20)_ = 0.48, *p* = 0.63.

**Figure 2:**
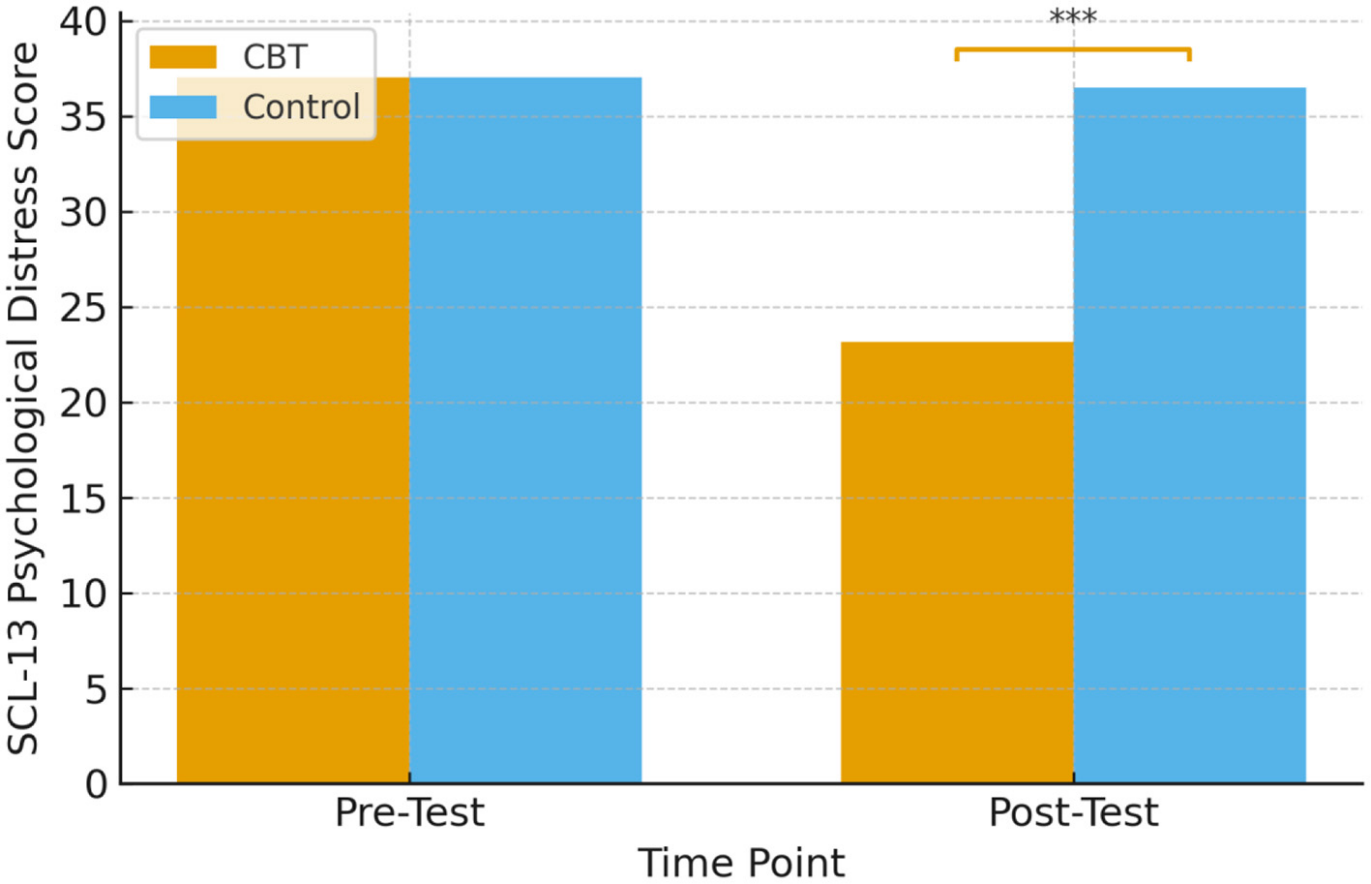
Bar chart comparing pre-test and post-test SCL-13 mean scores for the CBT and control groups. CBT, intervention; Control, waitlist/usual care. Error bars represent standard deviations. *p < 0.05; **p < 0.01; ***p < 0.001. Between-group differences at post-test were statistically significant (**p < 0.001). Visual Representation showing Between-Group Differences.

Between-group comparisons revealed a significant difference, with the CBT group showing lower post-test scores than the control group, *t*_(40)_ = 5.64, *p* < 0.001. These results indicate that participants in the CBT group had greater reductions in distress compared to those in the control group, who remained largely unchanged. The visual comparison of pre-test and post-test scores between the CBT and control groups is presented in Figure 2.

Because the severe and moderate groups started with different baseline levels of distress, comparisons were based on pre- and post-change scores and confirmed with an ANCOVA adjusted for age, rather than relying solely on post-test means.

Table 6 compares changes in psychological distress scores between the severe and moderate CBT intervention groups. Both subgroups experienced significant reductions in distress following the 8-week intervention. Severe participants improved from a baseline mean of 44.20 (SD = 3.10) to 28.10 (SD = 4.20) post-intervention, a reduction of 16.10 points, *t*_(9)_ = 5.85, *p* < 0.001, η^2^ = 0.62. Moderate participants improved from 30.50 (SD = 2.80) at baseline to 18.70 (SD = 3.00) at post-test, a reduction of 11.80 points, *t*_(10)_ = 4.42, *p* < 0.01, η^2^ = 0.48. Although both groups benefited substantially, ANCOVA adjusting for age indicated that reductions were greater in the severe group. This suggests that participants with more severe baseline distress experienced proportionally larger gains from CBT. These findings reinforce the overall effectiveness of CBT across severity levels, while highlighting potential added benefits for those with higher initial distress.

As shown in Table 7, participants in the CBT intervention groups demonstrated significant reductions in psychological distress by the midpoint of the programme. Severe participants improved from 44.20 (SD = 3.10) at baseline to 35.10 (SD = 3.80) at week 4, with a statistically significant change, *t*_(9)_ = 4.85, *p* < 0.001, η^2^ = 0.55. Similarly, moderate participants improved from 30.50 (SD = 2.80) at baseline to 24.50 (SD = 3.20) at week 4, with a significant change, *t*_(10)_ = 3.92, *p* = 0.003, η^2^ = 0.46. In contrast, no meaningful change was observed in the control group, where both severe and moderate participants maintained stable scores from week 1 to week 4 (*p* > 0.05). These midpoint findings suggest that CBT-related improvements began to emerge early, prior to the completion of the full 8-week programme.

**Table 7:** Paired *t*-tests of week-1 vs. week-4 scores (mid-intervention monitoring).

**Group**	***N***	**Week-1 M (SD)**	**Week-4 M (SD)**	**Mean Change**	***t(df)***	***p***	***η^2^***
Severe CBT	10	44.20 (3.10)	35.10 (3.80)	−9.10	4.85 (9)	< 0.001	0.55
Moderate CBT	11	30.50 (2.80)	24.50 (3.20)	−6.00	3.92 (10)	0.003	0.46
Severe control	10	43.50 (3.50)	43.80 (3.00)	+0.30	0.41 (9)	>0.05	–
Moderate control	11	31.20 (3.00)	29.90 (2.90)	−1.30	0.28 (10)	>0.05	–

Mid-intervention (Week 4) exploratory within-group comparisons of SCL-13 total scores (range: 0–52). Paired t-tests were used, with df = *n*−1. Week-4 analyses were secondary and served as an interim check, rather than replacing the primary outcomes measured at Week 8.

### Assumption testing

Before conducting parametric analyses, key statistical assumptions were examined to ensure the validity of the tests employed. The assumptions of normality and homogeneity of variance were tested prior to the analysis, and the results indicated that these assumptions were satisfied (Table 8).

**Table 8:** Statistical tests.

**Test**	**Group**	**Statistic**	***p*-value**	**Interpretation**
Shapiro–Wilk test (normality)	CBT group	0.97	0.68	Normality assumption met
Shapiro–Wilk test (normality)	Control group	0.98	0.83	Normality assumption met
Levene's test (homogeneity)	Both groups	1.4	0.24	Equal variances assumed (*p* > 0.05)

**Figure 3:**
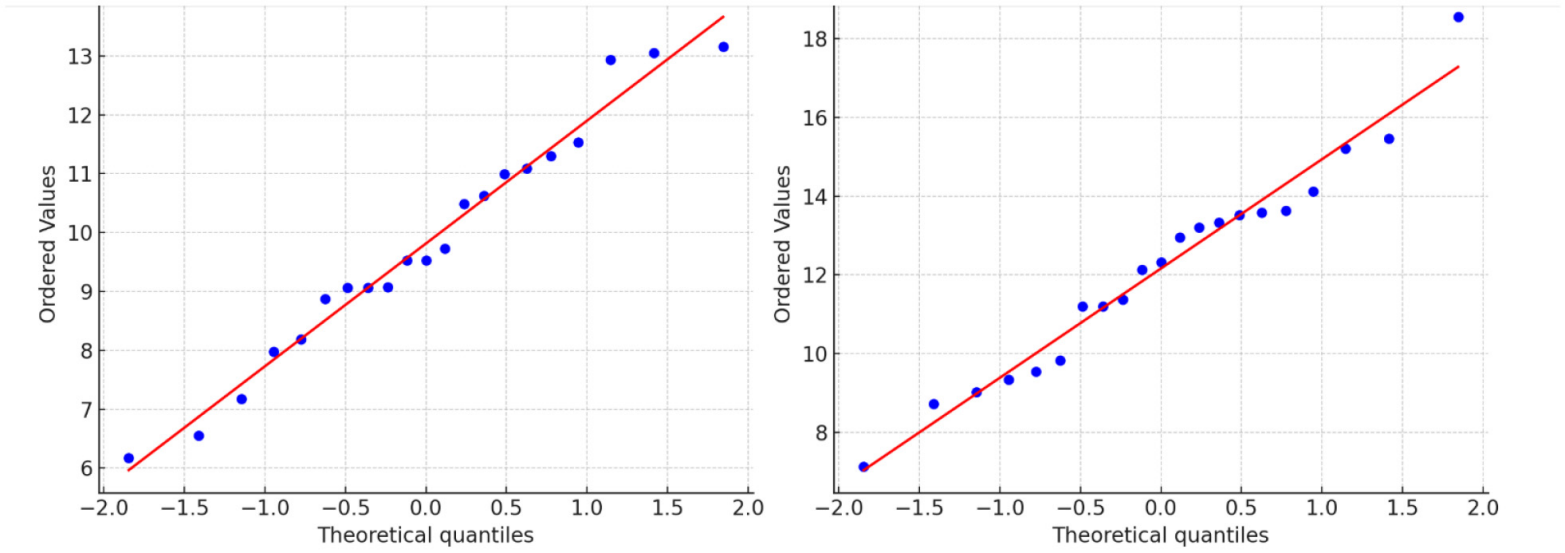
Outputs and interpretations based on the assumptions testing.

Normality of the data was assessed using the Shapiro-Wilk test and visual inspection of Q-Q plots (see Figure 3). Results indicated that psychological distress scores for both the CBT and control groups approximated a normal distribution (*p* > 0.05), justifying the use of parametric tests.

Homogeneity of variances was evaluated using Levene's Test for Equality of Variances. The test yielded non-significant results across pre- and post-test scores (*p* > 0.05), indicating equal variances between groups.

Collectively, these checks confirmed that the data met the assumptions required for conducting independent and paired samples *t*-tests, as well as Analysis of Covariance (ANCOVA), thereby supporting the robustness of the reported statistical findings.

### Q-Q plots

Q–Q plots of standardized residuals for the CBT and Control groups. The points lie approximately along the diagonal line, indicating that the data were normally distributed. These plots confirm the assumption of normality required for parametric analyses (paired and independent *t*-tests, ANCOVA). The ANCOVA results showed a statistically significant effect of the CBT intervention after controlling for baseline scores and sociodemographic variables (Table 9).

**Table 9:** Analysis of covariance (ANCOVA) results showing effectiveness of cognitive behavioral therapy intervention by sociodemographic characteristics among people living with hypertension.

**Source**	***df***	***F***	***p***	***Partial η^2^***
Age	(3, 38)	5.24	0.040	0.12
Gender	(1, 40)	1.99	0.164	0.05
Treatment	(1, 40)	14.30	0.001	0.26

CBT, Cognitive Behavioral Therapy; ANCOVA, Analysis of Covariance. A two-way ANCOVA examined post-intervention psychological distress scores, controlling for sociodemographic variables. All results are reported with two decimal places of precision.

**Hypothesis Two:** This hypothesis posits that Socio-demographic variables (age, gender, and marital status) will not significantly impact the effectiveness of CBT in managing psychological distress. The hypothesis was tested with an Analysis of Covariance (ANCOVA).

Analysis of covariance (ANCOVA) was performed to examine post-intervention differences, controlling for sociodemographic variables, to determine the effectiveness of CBT. Marital status was excluded from the final model due to its non-significance in preliminary analyses. The results indicated that age significantly affected treatment outcomes, *F*_(3, 38)_ = 5.24, *p* = 0.040, partial η^2^ = 0.115. In contrast, gender [*F*_(1, 40)_ = 1.99, *p* = 0.164, partial η^2^ = 0.047] was not a significant predictors. Importantly, treatment remained highly significant, *F*_(1, 40)_ = 14.30, *p* = 0.001, partial η^2^ = 0.263, confirming the effectiveness of CBT beyond the influence of socio-demographic variables.

## Discussion

This study aimed to determine the effectiveness of Cognitive Behavioral Therapy (CBT) in managing psychological distress in patients with high blood pressure, and to examine how sociodemographic variables influenced CBT outcomes. The study used a two-phase system, first evaluating individuals with hypertension in the clinic for psychological distress, followed by an 8-week cognitive behavioral therapy session to assess its effects. The results reinforce the use of CBT as a non-pharmacological, evidence-based psychotherapy for managing hypertension-related psychological distress, adding to the growing body of literature supporting psychological interventions in the management of long-term illnesses.

It was established that CBT produced important changes in psychological distress among individuals with hypertension, and the participants depicted a significant reduction in the degree of distress after the 8-week intervention program. This confirms the CBT efficacy regarding its applicability as an evidence-based and methodical psychological treatment system in the treatment of chronic conditions and associated emotional suffering. The role of CBT in hypertension has been supported in recent studies, including Li Y. et al. (2021) and Walker et al. (2024), who found that CBT-based interventions significantly lowered psychological symptoms and improved blood pressure control in patients with chronic conditions.

A meta-analysis by Magan et al. (2021) also identified CBT as a promising intervention that enhances the psychological wellbeing of patients with cardiovascular disease. However, some studies have raised doubts about the generalizability of CBT results among patients with hypertension. Notably, Tolin (2010) demonstrated that personal differences can mediate the effectiveness of CBT, particularly in relation to cognitive thinking and affective control. In the same vein, Li J. et al. (2021) demonstrated that mindfulness-based interventions can produce outcomes equivalent to, and at times superior to, those of CBT, suggesting that other psychological interventions may be equally effective in managing distress in individuals with chronic diseases. Such discrepancies in perceptions highlight the importance of an integrative approach to the treatment of people living with hypertension. In addition to usual medical care and psychological interventions, including CBT, other non-pharmacological interventions, such as relaxation training, guided imagery, and biofeedback, can be considered to optimize response to treatment.

The research concluded that age had a significant impact on the efficiency of CBT, as older participants reacted to the intervention differently from younger ones. In this way, it is suggested that cognitive and emotional processing may be altered by age, which can impact engagement with treatment and outcomes. The present finding can be supported by previous research conducted by Saunders et al. (2021), Werson et al. (2022), and Schneider et al. (2024), which suggests that older adults may require age-adapted CBT methods to receive maximal therapeutic benefit. On the other hand, the results of other researchers, including Granholm et al. (2013) and Salkovskis et al. (2023), indicate no significant difference in the outcomes of CBT among age groups; thus, it is possible that other factors, such as cognitive flexibility and motivation, may be more significant. Additionally, it is possible to enhance the effectiveness of CBT by incorporating age-related modifications, such as applying life review approaches to elderly adults or utilizing digital CBT for younger responders, and providing high-quality and high-proximity treatments.

Gender and marital status, on the other hand, had no significant effect on the results of CBT, meaning that the therapy was no more effective for any gender or marital status. It follows the results provided by Cuijpers et al. (2014) and Parisi et al. (2022), who state that gender is not a significant contributor to the responsiveness to CBT among patients with chronic illnesses. In the same way, Vaingankar et al. (2020) concluded that marital status exerted little effect on psychological interventions because social support networks other than the marital ones also influence the mental health outcomes. Nonetheless, not all studies have reported equally strong effects, with some showing more modest improvements. Factors like gender difference in emotional expression and futile coping styles (Lim et al., 2018) or marital illness that may moderate effects of the psychological interventions (Sanchis-Sanchis et al., 2020) were examples of factors that may make a difference in the effects of CBT. When these views are considered, incorporating additional supportive approaches, such as group therapy, social support systems, and culturally oriented counseling, may enhance the effectiveness of CBT across different demographic groups.

There is a strong rationale for incorporating psychological interventions in the management of hypertension, guided by the Cognitive Theory of Psychological Distress. Approaches such as CBT directly target maladaptive cognitions, while other non-pharmacological strategies, including mindfulness, meditation, relaxation training, and biofeedback, also aim to regulate affect through cognitive appraisal. Cognitive reappraisal is an important aspect of mindfulness that enables participants to reframe the emotional stimuli of the situation, thereby diminishing distress (Boroń-Krupińskaabde and Kulmatyckicde, 2016; Buhle et al., 2014; Carlson et al., 2025). Mindfulness and meditation are based on acceptance-related approaches that encourage emotional regulation by being non-judgmental of unpleasant experiences (Troy et al., 2018). On the one hand, CBT proved to be very effective in mitigating the psychological distress of those who were involved in it; however, it must also be noted that other non-pharmacological measures can be used alongside it or provide some other advantages. Mindfulness-based interventions, such as those, have demonstrated potential in distress management for chronic disease (Li J. et al., 2021), and additional studies are required to determine their relative efficacy in comparison to CBT in managing psychological distress induced by hypertension. They coincide with community-based interventions that focus on culturally responsive, holistic care that works on both the distress in the individual and the social determinants of health (Collins et al., 2018; Okunade et al., 2023). Hytman et al. (2025) observed that mindfulness programs delivered in a group and culturally adapted improve access and adherence to different populations. Combining CBT and a range of complementary and alternative medicine (CAM) practices in community environments could make hypertension management more inclusive, sustainable, and patient-centered.

In addition to the statistical gains being realized, there are several contextual issues worth considering. All the participants have been previously clinically diagnosed with hypertension and were recruited on ordinary clinic days. However, many presented with the “silent” type of hypertension and were largely asymptomatic at the time of enrolment. This absence of manifested physical symptoms minimized their perceived sense of urgency to receive psychological attention. It complicated the task of them being able to see the relationship between high blood pressure and emotional distress. However, upon enrollment, the participants acknowledged that CBT provided effective coping skills, which complemented medical care.

There was also engagement that was affected by the cultural elements. The degree of stigma against psychotherapy is quite high in Nigeria, especially when interventions are presented as psychiatric care. However, the situation appears to improve as psychological support is integrated into the treatment of chronic illnesses or provided in less stigmatizing settings, such as group formats. Recent evidence from research conducted in Nigerian hospitals suggests that integrating mental health care with physical health services reduces stigmatization and promotes engagement [World Health Organization, 2023b; International Cancer Control Partnership (ICCP), 2025]. A limited number of patients in this study declined CBT, and they were not assigned to the control group, so this minimized selection bias. These findings suggest that the impression of psychotherapy is gradually evolving as mental health becomes increasingly normalized in clinical contexts.

Although the 8-week intervention proved to have immediate positive effects, its sustainability is questionable in the long run. Longitudinal or stepped-wedge designs will be needed to establish whether CBT will alleviate distress and facilitate adherence in the long run. Notably, despite the intervention being carried out in a tertiary hospital with trained psychologists, the results prove that it is possible to implement psychological care as a part of hypertension management in Nigeria. CBT should be extended to primary care and community health centers, and this will need additional assessment. Nevertheless, the research paper points to its possible applicability to the general settings with low resources, where there is a shortage of mental health services.

## Implications for theory and practice

The results support the Cognitive Theory of Psychological Distress by showing that maladaptive appraisals and ineffective emotional regulation are factors that increase psychological burden in individuals living with hypertension and that can be effectively implemented through a structured CBT. The marked effect of age on treatment outcomes would indicate the possibility that the generational differences between groups, including cognitive flexibility, openness to therapy, and help-seeking behavior, would have an impact on how individuals respond, which would be crucial to age-sensitive modifications in the intervention design. Practically, the findings present the evidence that CBT could be introduced into the management of hypertension in Nigerian tertiary hospitals to meet the psychological demands alongside the use of pharmacological medication. Implementing CBT in chronic disease management processes can enhance adherence, patient satisfaction, and wellbeing, while serving as a model for expanding mental health services in other low-resource settings.

## Strengths and limitations

One of the strongest points of this study is that the concept of randomization and that of allocation concealment were achieved by way of the employment of an independently administered allocation list, which minimized the chances of selection bias. This strengthens the internal validity, as well as offers more rigorous evidence compared to quasi-experimental designs. It is also one of the earliest attempts at integrating CBT with hypertension care in Nigeria, thus adding valuable data on effectiveness. The use of validated instruments and detailed reporting of sociodemographic influences further strengthens its contribution to the evidence base.

Several limitations must be acknowledged. The time of intervention was relatively short, and conclusions regarding the long-term viability of the effects could not be drawn. Although both groups were invited to a formal debriefing session after the data collection period, which mitigated ethical concerns related to non-disclosure of support, the lack of longitudinal follow-up also indicated that there was no opportunity to assess any long-term psychological benefits. Nevertheless, the after-trial wellbeing was not guaranteed, and after the study time, participants were not provided with organized psychological assistance. CBT is not currently provided to the Obafemi Awolowo University Health Center (OAUHC); therefore, this study facilitated the implementation of CBT in the environment of the tertiary clinic to guide its future application. Although there are only a few patients who refused to participate, this may have caused some level of selection bias. They were analyzed on a per-protocol basis (only included those subjects who had full pre- and post-intervention data), which can overstate the treatment effects compared to an intention-to-treat (ITT) design. Although focus group discussions generated rich qualitative data on participants' experiences of CBT and hypertension care, detailed narrative or thematic analyses are beyond the scope of this paper and will be reported in a subsequent qualitative article. Lastly, the relatively small sample size, which limits the precision of estimates and the ability to detect small effects, and a hospital-based design decrease the generalizability to community environments. These constraints highlight the need for additional studies, including longitudinal follow-up, larger samples, and validation of scalable models of CBT in primary care and community settings. However, the selection of participants who refused to receive CBT might have led to selection bias because more willing participants to undergo psychotherapy might have been overrepresented in the sample.

## Conclusion

The participants who were given CBT showed a significant reduction in hypertension-related psychological distress, although some change was actually observed in the control group. The factor of age was found to be significant, compared to the factors of gender and marital status, indicating that treatment outcomes are age-sensitive. These results suggest that an 8-week CBT intervention can be successfully incorporated into hypertension management in Nigerian tertiary hospitals. However, longitudinal studies are necessary to demonstrate the long-term efficacy and sustainability of this approach. Future studies can also focus on the ways in which CBT can be supplemented with culturally acceptable methods, like mindfulness or relaxation training, to increase its acceptability and adoption. Generally, this study suggests that CBT can be integrated with a more holistic and patient-centered approach to chronic disease management, which encompasses both psychological and social aspects of care in Nigeria and other settings.

## Data Availability

The raw data supporting the conclusions of this article will be made available by the authors, without undue reservation.
